# An increase of phosphatidylcholines in follicular fluid implies attenuation of embryo quality on day 3 post-fertilization

**DOI:** 10.1186/s12915-021-01118-w

**Published:** 2021-09-09

**Authors:** Ju Wang, Wei Zheng, Shuoping Zhang, Keqiang Yan, Miao Jin, Huiling Hu, Zhen Ma, Fei Gong, Guangxiu Lu, Yan Ren, Liang Lin, Ge Lin, Liang Hu, Siqi Liu

**Affiliations:** 1 College of Life Sciences, University of Chinese Academy of Sciences, Beijing, 100049 China; 2 Clinical Reaseach center for Reproduction and Genetics in Hunan Province, Shenzhen, 518083 China; 3 BGI-Shenzhen, Changsha, 410008 China; 4 Institute of Reproductive and Stem Cell Engineering, School of Basic Medical Science, Key Laboratory of National Health and Family Planning Commission, Central South University, Changsha, 410008 China

**Keywords:** Assisted reproductive technology, Embryo quality, Oocyte quality, Targeted metabolomics, Follicular fluid, Random forest

## Abstract

**Background:**

Although oocyte quality is the dominant factor determining embryo quality, few studies have been conducted to evaluate embryo quality based on the metabolites related to the oocyte. With quantification of the follicular fluid (FF) metabolites, in assisted reproductive technology (ART), this study sought to evaluate the embryo or oocyte quality through an informative approach.

**Results:**

An evaluation model consisting of 17 features was generated to distinguish the embryo quality on day 3 post-fertilization, and phosphatidylcholines (PCs) were the key contributors to the evaluation. The model was extended to the patients under different ages and hyperstimulations, and the features were further enriched to facilitate the evaluation of the embryo quality. The metabolites were clustered through pathway analysis, leading to a hypothesis that accumulation of arachidonic acid induced by PCs might weaken embryo quality on day 3 post-fertilization.

**Conclusions:**

A discriminating model with metabolic features elicited from follicular fluid was established, which enabled the evaluation of the embryo or oocyte quality even under certain clinical conditions, and the increase of PCs in follicular fluid implies the attenuation of embryo quality on day 3 post-fertilization.

**Supplementary Information:**

The online version contains supplementary material available at 10.1186/s12915-021-01118-w.

## Background

In recent decades, assisted reproductive technology (ART) has been widely used in treating infertility [1, 2]. Preimplantation developmental arrest of embryos is a common phenomenon during the ART process, in which approximately half of in vitro produced embryos arrest during the first week of human embryogenesis [3, 4]. Oocyte quality is the major determinant of preimplantation embryo developmental competence [5, 6]. The oocyte delivers half of the chromosomal complement to the embryo and supplies many maternal factors to support the first three cell divisions during embryo development [7]. Oocyte quality can be evaluated through morphological observation in an in vitro fertilization (IVF) lab, whereas this technique is not satisfactory for clinical practice, due to the high frequency in identifying in a negative way [8]. An exploration of the sensitive operational indicators at the molecular level is urgently needed to facilitate the assessment of oocyte quality.

To have a better assessment, there are two fundamental questions, which examination is well acceptable in clinical to ensure oocyte quality and which material is clinically operational to get the quantitative information at a molecular level.

Some parameters as maturation, fertilization, and cleavage of oocytes have been gauged in the assessment of the oocyte quality in the clinical setting; however, the influence of these parameters on embryo quality is not predictable. In most in vitro fertilization-embryo transfer (IVF-ET) or intracytoplasmic sperm injection (ICSI) cases, the in vitro cultured embryo on day 3 is a final “product” whose quality is the most decisive factor that affects pregnancy, except for uterus status [9, 10]. More importantly, the embryo quality on day 3 post-fertilization is a well-accepted criterion in the clinical setting. As oocyte quality is positively correlated with embryo quality, the latter is believed to be representative of the former. Consequently, in vitro embryo quality on day 3 post-fertilization was assumed as a measurement of the oocyte quality in this study.

In a cycle of IVF-ET or ICSI, the cumulus-oocyte complex is first isolated from the individual’s follicular fluid (FF). The FF provides an essential microenvironment for oocyte development [11]; thus, it is reasonable and generally accepted that some molecules in the FF may be the indicators of oocyte quality [12–14]. Technically, the collection of FF in a noninvasive mode is easy and feasible in a clinical operation. The molecules in the FF are broadly divided into large molecules, with proteins being their main components, and small molecules, with most being metabolites. Although a proteomic approach was employed to find the protein biomarkers in the FF, a clinically applicable protein indicator is still unavailable because the high abundance of proteins in the FF hinders the discovery of the sensitive biomarkers [15–17]. Furthermore, the FF metabolites, including amino acids (AAs) [18, 19], hormones (HOs) [20–23], vitamins (Vits) [24–26], lipids [27, 28], and carbohydrates [29–31], have been considered indicative candidates of oocyte quality. However, none of them has become commonly accepted in clinical practice to date. Metabolomics has emerged as a powerful means of fully analyzing metabolites in bodily fluids, whereas the current profiling approach can provide limited information because it only enables semi-quantification and lower-level identification of compounds [32]. With the rapid development of targeted metabolomics, which offers satisfactory quantification of certain targeted chemicals, a combination strategy by integration of multiple targeted metabolomics was proposed in this study to discover the potential correlation between the FF metabolite quantity and oocyte quality [33, 34].

In this study, a total of 418 FFs were collected and multiple targeted metabolomics was employed to quantify the FF metabolites. By dividing the samples into two groups based on embryo quality on day 3 post-fertilization, the quantitative data were acquired for 136 metabolites from 8 metabolite groups, and machine learning was implemented to develop a random forest (RF) model that could discriminate embryo quality on day 3 post-fertilization at the metabolomics level. The RF model with 17 features was successfully developed, and under certain clinical conditions, RF models with fewer features were generated that could discriminate embryo quality on day 3 post-fertilization. For the first time, integrated targeted metabolomics was employed to demonstrate that some metabolites in FFs were significant indicators of embryo or oocyte quality.

## Results

### Generation of high-quality targeted metabolomics in FFs

Of the 418 FFs collected, 357 FFs were quantified for all targeted metabolomics analysis and 60 FFs were disqualified due to lack of entire information of target metabolomics. Limited by the available volumes of FF, the FF samples taken for individual targeted metabolome varied, and measurements were taken of P180, steroid HOs, water-soluble Vits, fat-soluble Vits, and AAs in 390, 417, 417, 417, 399, and 410 FF samples, respectively.

**Fig. 1:**
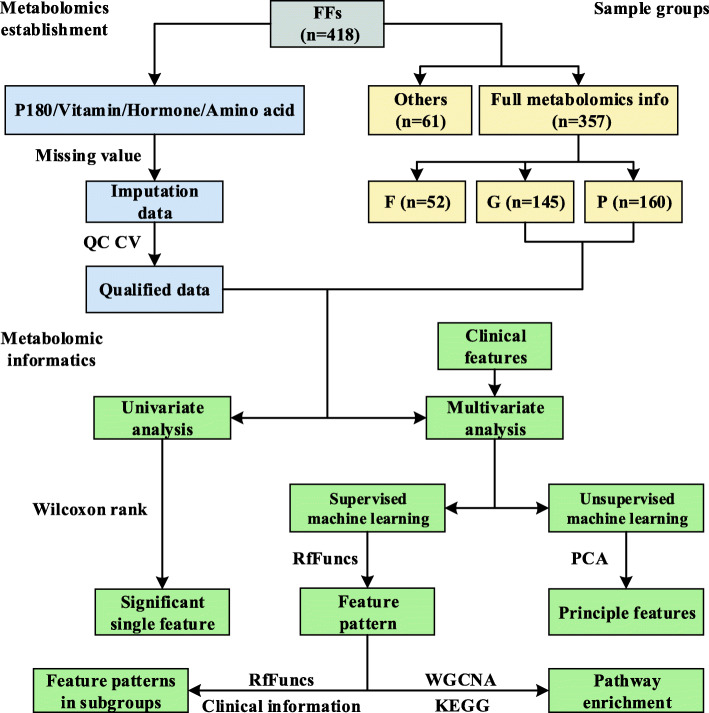
Workflow of the project design

**Fig. 2:**
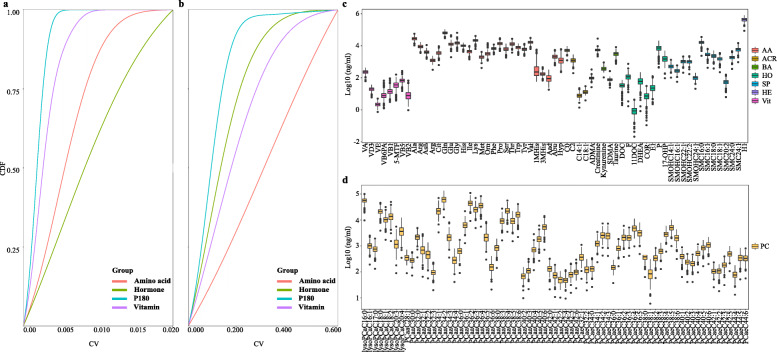
Data quality control for analysis of targeted metabolomics and the distribution of the metabolite concentrations in all the FFs. **A** The distribution of the cumulative frequency of the CV for the relative retention time in the QC samples. The *Y*-axis represents the cumulative frequency of the CV, and the *X*-axis denotes the CVs for the relative retention time. **B** The distribution of the cumulative frequency of the CV for the metabolite concentrations in the QC samples. The *Y*-axis represents the cumulative frequency, and the *X*-axis stands for the CV for the metabolite concentrations. **C**, **D** Concentration boxplots for all the targeted metabolites quantified in all the FFs. The *Y*-axis represents the concentrations (ng/ml) after log10 transformation, and the *X*-axis denotes the metabolites. AA, amino acid; ACR, acyl carnitine; BA, biological amine; HO, hormone; SP, sphingolipid; HE, hexose; Vit, water-soluble and fat-soluble vitamins; PC, phosphatidylcholine

The brief workflow of the data quality control (QC) and statistical analysis are shown in Fig. 1. A total of 214 metabolites were quantitatively targeted by triple quadrupole mass spectrometry (MS) with internal standards. Among the 214 metabolites, 70 metabolites were considered to be missing features. Therefore, only 144 metabolites were subsequently qualified. The coefficient of variance (CV) distributions of the relative retention time (analyte retention time/internal standard retention time (RRT)) in all the liquid chromatography with tandem mass spectrometry (LC-MS/MS) assays were quite stable. As shown in Fig. 2A, the average CVs of all the RRTs were between 0.08 and 0.56%. The CV distributions of the quantified metabolites in all the assays illustrated in Fig. 2B are largely acceptable due to the relatively narrow range of the average CVs, extending from 9.64 to 32.4%. With the cutoff in the quantification of 30% CV of QC samples and the MetIDQ criterion of P180, a total of 136 metabolites were qualified accordingly with no obvious batch effect (Additional file 1).

The boxplots of the metabolite concentrations in the FF are illustrated in Fig. 2C and D. In Fig. 2C, the Vits had the lowest concentrations, whereas the AAs exhibited the highest concentrations compared with the other metabolite groups. The metabolite concentrations for these targets in FFs generally appeared to have a large dynamic range. The ranges of the median concentration (RMC) of the Vits and biological amines (BAs) were relatively narrow, extending from 2 to 231 ng/ml and from 76 to 5449 ng/ml, respectively (Fig. 2C). Figure 2D displays the concentration distribution of all the phosphatidylcholines (PCs), and the RMCs of these PCs were not always consistent with the metabolites shown in Fig. 2C and had a large range, extending from 48 to 62430 ng/ml. Additionally, the RMCs of the HOs also had a wide scale of 0.9–7246 ng/ml. The relatively large concentration variations of the PCs and HOs in FFs might indicate that these metabolites are quite sensitive to the embryo status on day 3.

### Clinical information for the patients recruited in the study

As mentioned above, 357 patients qualified for further investigation, and they came from different medical backgrounds with varied physiological conditions, medical histories, statuses of the uterus and fallopian tubes, and controlled ovarian hyperstimulation (COH) protocols. These patients were divided into three groups—good (G), fair (F), and poor quality (P)—based on the “Methods” section, and the sample size was 145, 52, and 160, respectively. Simply by looking at the clinical parameters measured in the hospital, such as the rate of fertilization, and cleavage, no significant differences were found between groups P and G, except in 3 parameters: body mass index (BMI), retrieved oocytes (RON), and estradiol on the human chorionic gonadotropin injection day (Ie2). Importantly, these parameters, either alone or in combination, could not be successfully employed to predict embryo quality on day 3 post-fertilization (Additional file 2: Table.S1).

### Embryo qualities were well distinguished by the RF models derived from FF metabolic profiling

**Fig. 3:**
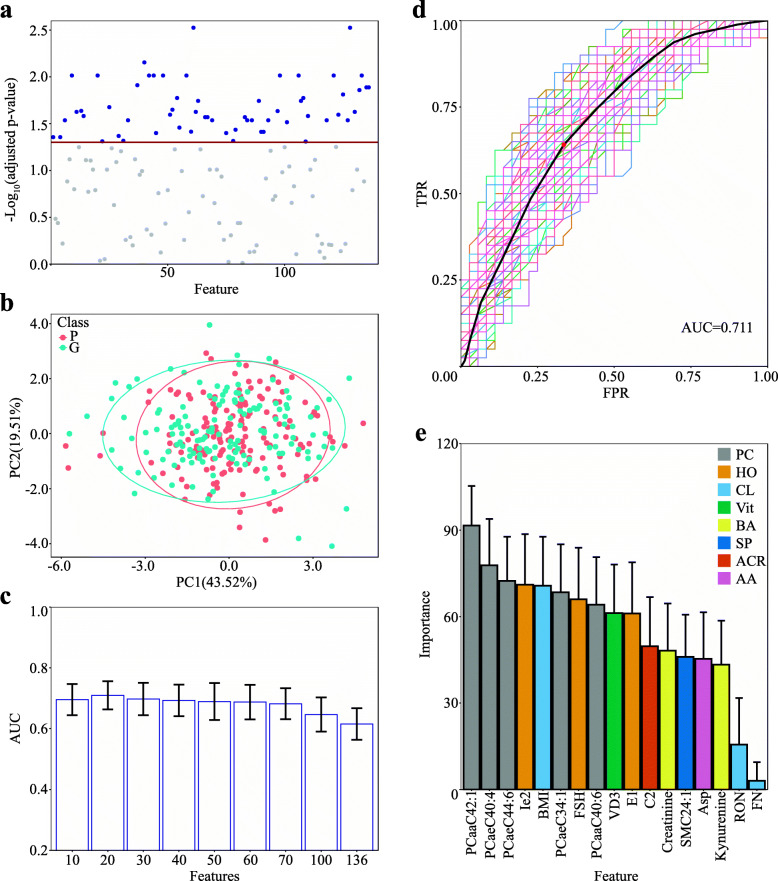
Statistical evaluation of the metabolomics indicators that can distinguish groups G and P. **A** Wilcoxon rank-sum test of the quantities of all the metabolites between groups G and P. Blue dots represent the metabolites with adjusted *p* values ≤ 0.05; gray dots stand for the metabolites with adjusted *p* values ≥ 0.05. The red line indicates the cutoff of the adjusted *p* value at 0.05. **B** PCA plot of the quantities of all the metabolites and some clinical parameters between groups G and P. The *X*-axis stands for the first principal component, and the *Y*-axis denotes the second principal component (G group, *n* = 145; *P* group, *n* = 160). **C** Dimensional reduction of the features consisting of the metabolites and clinical parameters with rfFuncs. The *Y*-axis represents the average AUC value of ROC in the RF models, the error bar represents the standard deviations of the AUCs, and the *X*-axis denotes the selected features. **D** ROC analysis of the RF model with 17 selected features upon 100 runs. The curves with color represent the ROC results predicted by the model running once. The black curve and the red dot represent the mean ROC curve and the optimal cutoff point, respectively. The *Y*-axis and *X*-axis stand for the values of the true positive rate and the false positive rate, respectively. **E** The averages of feature importance in the RF models with 17 selected features. The *Y*-axis represents the feature importance in the RF models, in which the error bars are the standard deviations elicited from the importance averages, and the *X*-axis stands for the features selected. AA, amino acid; ACR, acyl carnitine; BA, biological amine; CL, clinical feature; HO, hormone; PC, phosphatidylcholine; SP, sphingolipid; Vit, vitamin

Wilcoxon rank-sum tests were individually applied to the 136 quantified metabolites to explore whether the abundance of a metabolite in group G was significantly different from that in group P. In Fig. 3A, based on a criterion of adjusted *p* value < 0.05, 60 metabolites, including 44 PCs, 11 sphingolipids (SPs), 2 BAs, 1 HO, 1 Vit, and the sum of hexose, were regarded as the candidates whose abundances were significantly different between groups G and P. The abundance differences for most metabolites with significant changes between G and P were remained within a narrow range, abundance ratios (G/P) less than 1.2, and there were only two metabolites, Cortisone and PCaaC42:1, that had the ratio close to 1.2. Hence, univariate receiver operating characteristic curve (ROC) analysis was further employed for the individual metabolites to determine whether a metabolite abundance was able to differentiate the embryo qualities. The results of the ROC analysis are illustrated in Additional file 2: Table.S2 and reveal that all the metabolites had relatively smaller values of area under the curve (AUC), even though the AUC values of the top 10 metabolites were still less than 0.7. Comparing the results of the univariate analysis obtained from the Wilcoxon rank-sum tests and the ROC reveals that these two approaches reached a similar conclusion and that none of the 136 metabolites would be a unique factor to distinguish the FFs derived from groups G and P. Additionally, all the top 10 metabolites with higher AUC values were categorized in the PC group. To better distinguish groups G and P, multivariable analysis was further applied in the following informatics analysis.

Under unsupervised conditions, principal component analysis (PCA) was implemented in multivariate analysis. In this PCA, 60% of the 136 targeted metabolites plus 21 clinical parameters were regarded as the principal components; however, the two groups, G and P, were not well separated by these components (Fig. 3B). This implied that a simple application of multivariate analysis without supervision to a relatively small cohort was not sufficient to distinguish groups G and P. Therefore, rfFuncs, a recursive feature elimination algorithm based on RF, was employed to seek another efficient solution under the supervised conditions. By running it 1000 times against a random 90% of the cohort as a training set and setting the remained features as 10, 20, 30, 40, 50, 60, 70, and 100, the optimal RF models were built, and the corresponding AUC values were estimated, as shown in Fig. 3C. The highest average AUC appeared in the group of 20 selected features, while the AUC values with more than 70 selected features were clearly lower. To better select the feature combination for distinguishing the embryo quality on day 3 post-fertilization, rfFuncs was rerun another 1000 times against the cohort by selecting groups of 5 to 25 features, revealing that 17 features were the best combination with the highest AUC in all the selected groups (Additional file 3). The next question was which feature patterns were representative for all the 17 feature combinations. By randomly selecting the 17 features, rfFuncs was again run against the cohort, and the frequencies of each feature in the 17 feature combinations were regarded as their potentials of being involved in the final pattern of 17 features. Based on the feature frequencies in Additional file 4, the final feature pattern was determined to consist of 5 PCs, 3 HOs, 2 BAs, 1 acyl carnitine (ACR), 1 VI, 1 SP, 1 AA, and 3 clinical parameters. With this feature pattern, the RF models were generated by running 100 times, accompanying the average ROCs with an average AUC of 0.711, sensitivity of 0.641, and specificity of 0.665 with a cutoff value of 0.55 (Fig. 3D). It is generally accepted that AUC values equal to or greater than 0.7 would be satisfactory in a discrimination model; therefore, the RF analysis indicated that the pattern with 17 features enabled discrimination of embryo quality on day 3 post-fertilization.

In the RF model, feature importance represents the extent of the contribution of a feature to the model, with a higher score indicating the greater contribution of a feature. The feature importance of the 17 features above, including 14 metabolites and 3 physiological features, is illustrated in Fig. 3E, and the top three important features were all PCs: PCaaC42:1, PCaeC42:6, and PCaeC44:6. Among the physiological features, BMI contributed a middle-level influence on the model, whereas the RON and the follicle number (FN) were ranked at the least importance. Examining the biophysical parameters of the 14 metabolites, approximately 70% of them possessed higher hydrophobic scores. Among these metabolites, the PCs, VD3, Ie2, E1, and SMC42:1 were ranked at relatively higher importance, whereas the remaining 5 hydrophilic metabolites were shown to be of relatively less importance. In summary, the dominant metabolites with higher hydrophobicity in the FF were likely to exert more influence to distinguish good and poor embryos.

### Discrimination model of embryo quality on day 3 post-fertilization worked appropriately for patients under certain clinical conditions

It is well accepted that the two clinical conditions, age and COH protocol, may influence oocyte quality. To reduce the factors involved in clinical diversity, therefore, the patients were further grouped into 5 subgroups based on the two conditions. All the condition details are illustrated in Additional file 2: Table.S3, in which more than 70 FF samples were collected under a given condition, while the sample ratios of G/P were approximately 0.8 to 1.2. With the feature pattern described above, RF models were generated by 100 runs for the FF samples under certain conditions. Additional file 5 presents the corresponding average ROCs accompanying the average AUCs and the importance of the 17 features to the models. The average AUCs under most conditions, with the exception of the long protocol, were greater than 0.7, indicated that even under these clinical conditions, the feature patterns were favorable to distinguish the embryo quality on day 3 post-fertilization. However, the figures on the lower panel of Additional file 5 presented the pertinent information, in which although the feature pattern could perform the good prediction in most subgroups with AUC higher than 0.7, the feature importance under different clinical conditions was diverse. For instance, in the FFs collected from women whose ages were equal to or greater than 35, the 4 PCs appeared to be the most important of the 17 features, whereas in samples from women younger than 35, only 1 PC was ranked at top importance. This raises the question of whether some FF metabolites might be more closely associated with the embryo quality on day 3 post-fertilization under certain clinical conditions.

**Fig. 4:**
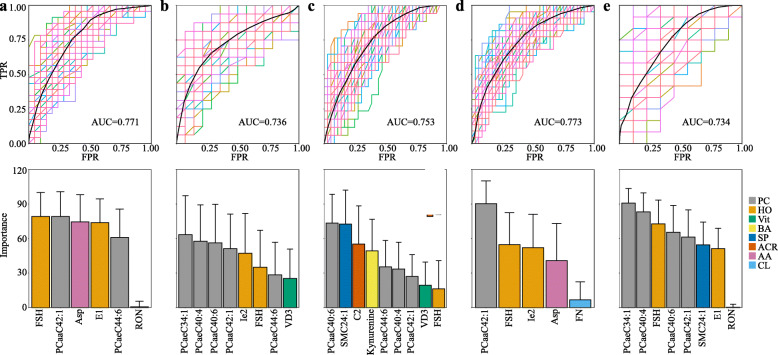
Discrimination of embryo quality based on the metabolomic indicators with clinical parameters. Discrimination is based on 2 lines of statistical evidence. The upper panel shows the ROC analysis, and the lower panel shows the averages of feature importance in the RF model. A total of 5 subgroups of the clinical parameters are selected: **A** the patients aged < 35, **B** the patients aged ≥ 35, **C** the patients who received the long protocol, **D** the patients who received the ultralong protocol, and **E** the patients who received the GnRH-ant protocol. The *X*-axis and *Y*-axis represent the same variables as in Fig. 3D and E

RFfuncs was rerun with the 17 features for the FFs under certain clinical conditions, and the satisfactory RF models with AUCs greater than 0.7 able to distinguish groups G and P were able to reach the optimal values with only 5 to 9 features (Additional file 6). For the patients with age < 35 and age ≥ 35, the optimal features were reduced to 6 and 8, respectively (Fig. 4A, B). For the feature importance, PCs and HOs were still the major contributors to the recognition of groups G and P for both age groups. However, in contrast to the feature importance in the total cases in this study (Fig. 3E), the importance of Asp in the features of the age < 35 group was ranked at a higher level. For the patients treated with different COH protocols, the optimal features decreased to 9, 5, and 8 for the long, ultralong, and GnRH-ant protocol groups, respectively (Fig. 4C–E). Regarding the feature importance, HOs and PCs were still estimated as the dominant influences on the discrimination models in the patients treated with the ultralong and GnRH-ant protocols. The feature importance for the long protocol group, nevertheless, appeared in an entirely different order, with the importance of SM24:1, C2, and Kynurenine increasing, whereas the influence of HOs was reduced. Examining all the information described in Fig. 4A–E, under specific clinical conditions, the metabolomics features that served to distinguish embryo quality on day 3 post-fertilization could be further concentrated from among the 17 features, and moreover, the concentrated features were not generalizable but were specific to certain conditions, in either features or importance.

### Oxidative stress in the FF induced by PC accumulation was an indicator of embryo quality on day 3 post-fertilization

**Fig. 5:**
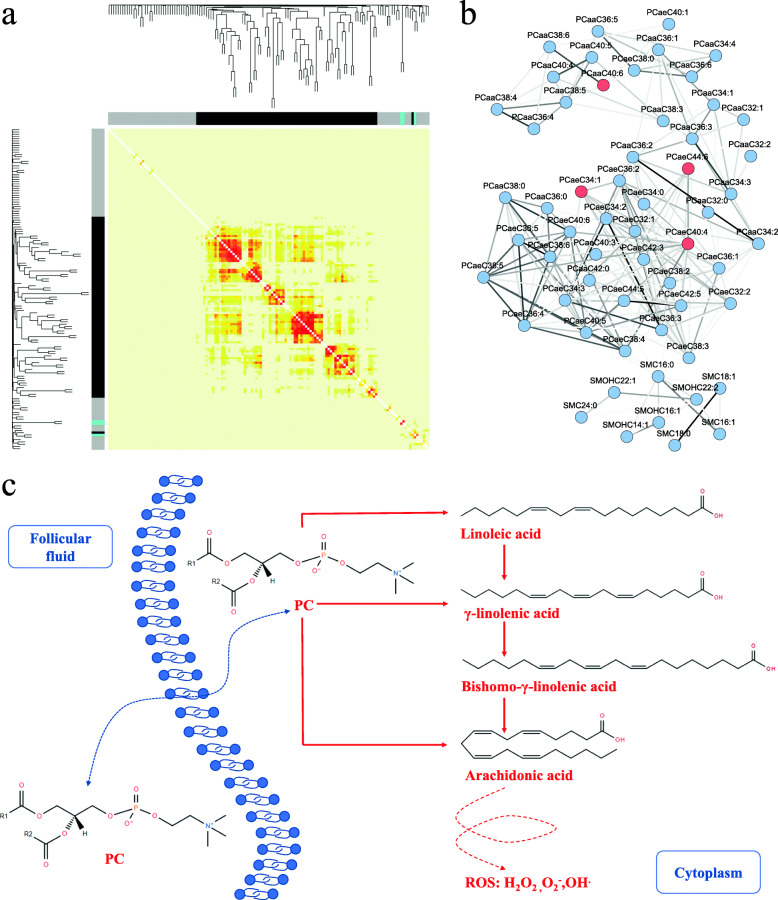
Network of metabolite interactions derived from WGCNA and the potential mechanism proposed from the targeted metabolomics. **A** WGCNA on the targeted metabolomics. The clustering dendrogram generated from the pairwise similarities of the metabolites resulted in 3 modules, gray, black, and cyan. The metabolite network is visualized by a heatmap that depicts the topological overlap matrix among all the metabolites, with yellow for dissimilarity and red for similarity. **B** The network nodes of the metabolite interactions derived from the black module of the WGCNA. The red nodes represent the PCs in the 17 features pattern. **C** A hypothetical model from the metabolomics indicators and the interaction network related to the PCs

To determine the relationship between metabolomics and embryo quality on day 3 post-fertilization, the 142 metabolites were loaded into weighted gene co-expression network analysis (WGCNA), resulting in these metabolites being clustered into 3 modules, with the black module containing 81 metabolites, the gray having 5, and the cyan possessing 3 (Fig. 5A). According to the WGCNA modules, the 14 metabolomics features responsible for the embryo quality discrimination model were located in two modules, that is, 6 in black and 8 in gray. The module acceptance was further evaluated by the topological overlap matrix (TOM), which revealed that the feature correlations in the black module were significantly accepted, but the others were not (Fig. 5A).

The black module was thus selected to build the metabolites network. Based on the metabolite adjacency matrix, the TOM analysis further revealed the potential network of the metabolite interactions in the black module. In the network shown in Fig. 5B, most PCs and SPs were involved, especially the 4 PCs derived from the 14 metabolomics features. As indicated in Fig. 3E, the 5 PCs in the FFs could greatly help to distinguish good and poor embryos, and their abundances in group P were significantly greater than those in group G. Furthermore, the contents of all the PCs in the FFs of group P were generally greater than those in G, as shown in Additional file 2: Table.S4. The accumulation and the pathway involvement of the PCs in the FFs were thus deduced to have a negative effect on the embryo quality on day 3 post-fertilization.

Since PCs occupied almost 85% of the metabolites in the black module and the chemical structures of these PCs were highly similar, it was difficult to statistically evaluate the pathway enrichment. All the PCs in the black module were broadly categorized as PCaa, PCae, or lysoPC. As the lysoPCs were filtered out due to the filtration of the adjacency matrix in the black module (Fig. 5B), the remaining PCaas and PCaes were used for the pathway analysis. According to the pathway annotation in the Kyoto Encyclopedia of Genes and Genomes (KEGG), the two kinds of PCs were simply merged into 2 pathways, arachidonic acid and linoleic acid metabolism, in which the PCs could be catabolized to fatty acids with long chains of alkane or alkene that further served for the synthesis of arachidonic acid. Since PCs are a chemical class with strong hydrophobicity and no membrane barrier for transportation in and out of cells, it is predictable that the increased concentration of PCs in the FF may partially reflect the PC abundance in the oocyte. A model of the functions of the accumulated PCs and arachidonic acid in the oocytes and FFs of the P group is hypothesized in Fig. 5C, in which the increased PCs in both the oocytes and FFs could lead to the generation of more arachidonic acid, which is an important source of reactive oxygen species (ROS) and is likely to strengthen the oxidative stress within the oocytes.

## Discussion

Many endeavors have been devoted to assessing embryo quality even through metabolic indicators regardless of in vivo or in vitro [35, 36]. The primary questions thus naturally go to which new information for evaluating embryo or oocyte quality should be gained from this study and what is a unique consideration in this field that is so different from the previous reports. First of all, how did we find a proper angle to utilize the metabolomic information for analyzing embryo quality on day 3 post-fertilization? It is generally accepted that oocyte is a key factor to determine the quality of the embryo, and the metabolites in FFs could partially reflect the oocyte living state [9, 10, 12–14]. A disturbing question is that the evaluation towards oocyte derived from current techniques in clinical is still less convincing to predict the embryo quality on day 3 post-fertilization, while the FF’s biochemical features seem a close association with oocyte but not with the embryo. Which association between the FF’s metabolomics and embryo quality on day 3 post-fertilization should be reasonably evaluated? We proposed that as an embryo on day 3 over grade 7C-II under a microscope is generally accepted in good quality, the corresponding oocyte should be reasoned as a good one as well. The embryo quality on day 3 post-fertilization assessed by clinical was thus assumed a representative of oocyte quality. The assumption brought two advantages, a clear definition for the quality of oocyte and embryo, and a logical connection between oocyte and metabolites in FF. Secondly, the metabolomics survey in FFs in this study should be deemed monitoring the FF metabolites at a large scale, and identifying and quantifying the metabolites in high quality. The profiling approach in metabolomics is limited in the high annotation of MS signals for metabolites, while the targeting method is restricted in less metabolites in a survey [33, 34]. We hence made an assessment strategy that integrated a number of targeted metabolites, which would be identification and quantification in good data quality. As a matter of fact, a total of 214 identified and quantified metabolites were satisfied by statistical estimation towards over 300 FFs. Thirdly, the large dataset with FF samples over 300 and the metabolites identified over 100 laid down a puzzle how to explore the indicative metabolites related with good or poor quality of embryo. In contrast to a traditional target study with a few of indicative features, the metabolomic survey here sought an indicator panel with some metabolites from a large candidate pool. Machine learning approach plays a key role to define the metabolic panel that contains the primary features with sensitive discrimination of embryo quality on day 3 post-fertilization. As demonstrated in Figs. 3 and 4, the panel with 17 features could not only recognize the embryos in good quality but also exert such recognition under certain clinical conditions. Gathering all the considerations above, the targeted metabolomics of the FFs is persuasively delivered unique information in evaluation of embryo quality on day 3 post-fertilization.

Of the features of the discrimination model, PCs offer an important contribution towards both occupation and importance. As the structural units on plasma membranes, PCs have been found many involvements in oocytes or embryos and the ART-related issues. For the embryo cryopreservation in ART, PCs were regarded as potential markers of Nellore and Simmental embryos in either in vitro or in vivo [37]. For searching diagnostic biomarkers of ovarian endometriosis, the elevated abundance of 8 lipid metabolites consisting of 5 PCs and 3 SPs was perceived as a close association with endometriosis [38]. Fatty acids are the sources and metabolite products of PCs and have been reported the special roles in oocytes and early embryos. For instance, Wallace claimed 9 fatty acids in the FFs of cleaved samples significantly different from that in the non-cleaved samples [39], while Marei observed linoleic acid, a major fatty acid in bovine FFs, to exert a reversible inhibitory effect on oocyte maturation in vitro [40]. Using the state-of-the-art technology, metabolomics towards oocyte and FF reached a similar conclusion PC and/or fatty acids as indicative molecules. A lipidome revealed that lipid and phospholipid species as the biomarkers of oocyte developmental competence and of follicular and oocyte aging [41, 42]. With only 6 lipids acquired from FF lipidome, the lipid panel was regarded a measurement of oocyte development and embryo implantation [43]. The results gained from the targeted metabolomics were basically in agreement with other observations that the PC’s importance in FFs could potentially guide the selection of a good oocyte or embryo. Moreover, our data described above provided the solid MS/MS evidence in both clearly identified PC molecules and accurately quantitative evaluation.

It is well known that PCs are the major phospholipid species on eukaryotic cell membranes, while PCs also function as a major source of intracellular signaling molecules. Hydrolysis of PCs by either phospholipase C or the combined action of phospholipase D and phosphatidate phosphohydrolase results in diacylglycerol, then produces fatty acids in a cell, arachidonic acid as the primary fatty acid [44]. Once the metabolic system of PCs is activated, the levels of diacylglycerol and fatty acids are elevated, leading to the generation of free radicals and hydroperoxides [45]. On the other hand, reacylation of diacylglycerol and fatty acids to phospholipids requires ATP that is made by oxidative metabolism; therefore, accumulation of PC metabolites within a cell would cause a deficiency of oxygen delivery. The targeted metabolomics here demonstrated a significant increase of PCs in FFs, while these accumulated metabolites in FFs were reasoned to be released from the oocytes and to reflect the PC levels within oocytes. Based on the experimental data and the theory of PC metabolomics, hence we made a hypothesis as shown in Fig. 5C, in which the PC accumulation might trigger an increase of oxidative stress within oocytes. The hypothesis is not only elicited from our observation but is also endorsed by others. Arachidonic acid was scrutinized in many issues related with oocyte fate, such as oocyte maturity [46, 47], meiotic resumption [48, 49], ovulation [50, 51], and fertilization [52]. Ciepiela pointed out that the FFs of oocytes with the disappearance of two pronuclei or degeneration after ICSI exhibited the increased activity of secretory PLA2 and significantly higher arachidonic acid derivatives [52]. In contrast to the early investigation, we do not stop at the simple observation that the FF’s PCs could sensitively discriminate the embryo with good or poor quality, but want to extend a theoretical model that explains possible disturbance of PC metabolism in oocytes.

The evaluation of oocyte/embryo quality upon target metabolomics in FFs was still at a preliminary stage. First of all, the conclusion was only drawn from the metabolomics measurements to the patients from a hospital, which is located at the middle of China. Whether the data of target metabolomics in FFs is dependent on the local population or different nationalities has been not systemically evaluated yet. Secondly, as mentioned above, morphologic observation to embryo quality on day 3 post-fertilization is a general approach accepted by the hospital; however, many clinical parameters potentially useful for evaluation of embryo quality are open questions for this field, such as embryo at day 5, implantation rate, heart-beat, and birth rate. Thirdly, a metabolomic feature consisting of several metabolites as a prediction factor to embryo quality on day 3 post-fertilization is only limited on the experimental stage and is unlikely to be a clinical approach. Optimization of metabolite measurement and refining of these selected metabolites, therefore, are necessary in the next study.

## Conclusions

An evaluation model consisting of 17 features from the target metabolomics in FFs was generated to distinguish the embryo quality, in which phosphatidylcholines were the key contributors for the discriminator. The model was extended to the patients under different ages and hyperstimulations, and the features were further enriched to facilitate the evaluation of embryo quality. The metabolites were clustered through pathway analysis, leading to a hypothesis that accumulation of arachidonic acid induced by phosphatidylcholines might affect embryo quality.

## Methods

### Evaluation of embryo quality on day 3 and collection of FFs

All the FF samples were donated from 418 patients who provided informed consent at the Reproductive and Genetic Hospital of CITIC-XIANGYA, China. This study was approved by the Ethics Committee of the Reproductive and Genetic Hospital of CITIC-XIANGYA (reference LL-SC-2018-036) and Beijing Genomics Institute (BGI) (reference BGI-IRG 20035). The oocytes were retrieved transnationally under ultrasound guidance in routine-assisted reproductive therapy. The transparent FF was collected and stored at −80°C until further metabolic analysis. The consequent embryos on day 3 after oocyte retrieval were evaluated upon the Puissant scoring system with slight modifications [53]. A high-grade embryo should be scored at grade >7C-II, in which the number of blastomeres was more than seven and fragmentation was less than 10% to the total blastomeres size, while a low-grade embryo should be scored at grade < 6C-II, in which the number of blastomeres was less than six as well as those embryos with more than 50% fragmentation. The representative morphologic figures of the embryos with high grade (grade >7C-II) and low grade (grade < 6C-II) were shown in Additional file 7. These patients were divided into three groups—good (G), poor (F), and fair quality (P). If all the embryos in a patient on day 3 were observed as high grade, the patient was placed into group G. If all the embryos in a patient on day 3 were observed as low grade, the patient was placed into group P. If a patient possessed some embryos with high grade while some with low grade, such patients were classified as group F.

### Sample preparation for targeted metabolomics

All the collected FFs were thoroughly vortexed after thawing and centrifuged at 4°C for 5 min at 5000 g. The supernatants were directly used for metabolite extraction. According to the manufacturer’s user guide, the 188 metabolites were extracted from the FFs by Biocrates® Absolute IDQ P180 kit (Biocrates Life sciences AG, Innsbruck, Austria). An aliquot of FF placed on the membrane of a 96-well microplate was fixed with phenyl isothiocyanate for derivatization, and the derivative products were eluted by 5 mM ammonium acetate in methanol. For the 5 fat-soluble Vits, the solution of methanol/acetonitrile (*v*/*v* = 1:1) was mixed with FFs on a 96-well plate, and the resulting supernatant was taken for further analysis after vortex and centrifugation. For the 5 water-soluble Vits, the FFs were mixed with a solution of methanol/water (*v*/*v* = 9:1), followed by vortex and centrifugation to obtain the supernatant. The steroid HOs were extracted by methylene chloride, dried with nitrogen, and resolved in methanol/water (*v*/*v* = 1:3). For AAs, 0.1 g/ml sulfosalicylic acid was added to the FFs for protein precipitation, and the supernatant after centrifugation at 4000 rpm was directly loaded into UPLC.

### LC-MS/MS analysis for targeted metabolomics

Three types of triple quadrupole mass spectrometers were adopted for targeted metabolomics, Xevo TQ-S (Waters, Milford, USA) for fat- and water-soluble Vits, QTRAP 5500 mass spectrometer (SCIEX, Villebon-sur-Yvette, France) for AAs, and steroid HOs and QTRAP 4500 mass spectrometer (SCIEX, Villebon-sur-Yvette, France) for the P180 kit. The MS parameters for each machine were optimized during the experiments, and the *M*/*Z* values of the parent/daughter ions in MRM mode were recommended by the reagent supplier or experiments. Most detections to the charged ions were set at positive mode except hexose (HE) at negative mode. The information regarding the separation of all the metabolites is listed in Additional file 2: Table.S5.

### Data quality control and preprocessing for MS/MS data

The quality control samples, either provided by a commercial source or prepared from extracts of the pooled FFs, were alternatively placed every 16–19 samples during the experiment using a mass spectrometer. Whether the MS/MS data were accepted for further informatics analysis was based on the data quality control criteria, either Biocrates rules for the P180 kit or CV less than 30% in our laboratory [54]. PCA with MetaboAnalyst version 4.0 (https://www.metaboanalyst.ca) was applied to evaluate the batch effect caused by the parallel experiments. According to the “80% rule,” a feature was regarded as a missing value if it was not detected in less than 80% of samples [55]. The missing values were treated with the imputation mode, in which the missing values caused by nonrandom processes were imputed with half of the minimum value in all measurements, and the missing values resulting from random processes were filled with the k-nearest neighbors using MetaboAnalyst version 4.0. For better multivariate analysis, all the features were run with centralization to make them comparable.

### Statistical analysis

To evaluate which FF metabolites were significantly different between the different groups, univariate analysis of the targeted metabolomics data was processed with Wilcoxon rank-sum test using MetaboAnalyst 4.0. For the same purpose, multivariate analysis was implemented with two approaches, unsupervised and supervised. Under the unsupervised condition, PCA was employed using the prcomp package to explore which metabolites primarily contributed to distinguish the different groups. Under the supervised condition, rfFuncs, an algorithm based on RF and written for R with the Caret package, was used to reduce the dimensions of the features. The optimal iteration runs to select the features were first optimized from 50 to 2000, then the lowest runs at the saturation phase were set as the optimal one, 1000 in this study. With 10-fold cross-validation repeated 3 times, RF models were built with the feature pattern recommended by rfFuncs to discriminate the different groups. Corresponding test sets were evaluated with ROC analysis, and AUC in ROC was a decisive parameter to judge whether a prediction was acceptable. Two-tailed statistical analysis was applied in all the involved tests.

With the analysis of WGCNA using R, all the targeted metabolites were grouped into several modules based on their abundance accordance. Furthermore, the TOM was employed to estimate the correlation efficiency of the two metabolites in a module and to deeply examine a potential network of the metabolites in such a module. Finally, the metabolites in the network overlapped with those in the prediction model, and the overlapped metabolites were inputted into KEGG database (https://www.genome.jp/kegg).

## Supplementary Information

**Additional file 1:.** Assessment of batch effect with PCA plot in each LC-MS/MS method. The batch effect of amino acid, fat-soluble vitamin, water-soluble vitamin, hormone and P180 method is represented in a-e, respectively.

**Additional file 2: Table.S1** Clinical information of the patients. **Table.S2** The top 10 AUCs and the corresponding optimal specificities and sensitivities of the metabolites with significant difference between G and P. **Table.S3** The sample size in different subgroups. **Table.S4** Fold changes of PCs with significant difference between G and P. **Table.S5** Elution gradient of all the UPLC methods.

**Additional file 3:.** Further dimensional reduction of the features consisting of metabolites and clinical parameters rfFuncs. Y-axis represents the average AUC value of ROC in RF models, error bar represents the standard derivation of AUCs, and X-axis denotes the selected features.

**Additional file 4:.** The frequency of metabolites selected in 17 features pattern.

**Additional file 5: **A discrimination is relied on 2 statistical evidences, upper panel, ROC analysis, and lower panel, averages of feature importance in a RF model with 17 features. A total of 7 subgroups upon the clinical parameters are selected, (**A**) the patient ages < 35, (**B**) the patient ages ≥ 35, (**C**) the patients received long protocol, (**D**) the patients received ultralong protocol, (**E)** the patients received GnRH-ant protocol. The representativeness of X-axis and Y-axis is as same as Figure 3D and E.

**Additional file 6: **Further dimensional reduction of the features consisting of metabolites and clinical parameters with rfFuncs in each subgroup. A total of 7 subgroups upon the clinical parameters are selected, (**A**) the patient ages < 35, (**B**) the patient ages ≥ 35, (**C**) the patients received long protocol, (**D**) the patients received ultralong protocol, (**E**) the patients received GnRH-ant protocol. The representativeness of X-axis and Y-axis is as same as Figure 3C.

**Additional file 7: **The morphology of the embryos on day 3 after fertilization. (**A**) The morphology of the embryo evaluated as G group on day 3 after fertilization (8C-I); (**B**) The morphology of the embryo evaluated as P group on day 3 after fertilization (IV).

## Data Availability

All the corresponding information of the research is available through the MetaboLights (https://www.ebi.ac.uk/metabolights/) with accession number MTBLS1915 and China National GeneBank Sequence Archive (CNSA) (https://db.cngb.org/cnsa/) with accession number CNP0001558. The major code with R is available on GitHub with the link, https://github.com/wangjums/FF-metabolomics.

## Abbreviations

FF: Follicular fluid
ART: Assisted reproductive technology
PC: Phosphatidylcholine
IVF: In vitro fertilization
ICSI: Intracytoplasmic sperm injection
IVF-ET: In vitro fertilization-embryo transfer
AA: Amino acid
HO: Hormone
Vit: Vitamin
RF: Random forest
MS: Mass spectrometry
RRT: Relative retention time
LC-MS/MS: Liquid chromatography with tandem mass spectrometry
RMC: Ranges of the median concentration
BA: Biological amine
COH: Controlled ovarian hyperstimulation
BMI: Body mass index
RON: Retrieved oocytes
Ie2: Estradiol on the human chorionic gonadotropin injection day
SP: Sphingolipid
ACR: Acyl carnitine
FN: Follicle number
TOM: Topological overlap matrix
KEGG: Kyoto Encyclopedia of Genes and Genomes
ROS: Reactive oxygen species
QC: Quality control
CV: Coefficient of variance
PCA: Principal component analysis
ROC: Receiver operating characteristic curve
AUC: Area under curve
WGCNA: Weighted gene co-expression network analysis
HE: Hexose
