# Publisher Correction to: An increase of phosphatidylcholines in follicular fluid implies attenuation of embryo quality on day 3 post-fertilization

**DOI:** 10.1186/s12915-021-01175-1

**Published:** 2021-11-02

**Authors:** Ju Wang, Wei Zheng, Shuoping Zhang, Keqiang Yan, Miao Jin, Huiling Hu, Zhen Ma, Fei Gong, Guangxiu Lu, Yan Ren, Liang Lin, Ge Lin, Liang Hu, Siqi Liu

**Affiliations:** 1 College of Life Sciences, University of Chinese Academy of Sciences, Beijing, 100049 China; 2 Clinical Research Center for Reproduction and Genetics in Hunan Province, Reproductive and Genetic Hospital of CITIC-XIANGYA, Changsha, 410008 China; 3 BGI-Shenzhen, Shenzhen, 518083 China; 4 Institute of Reproductive and Stem Cell Engineering, School of Basic Medical Science, Key Laboratory of National Health and Family Planning Commission, Central South University, Changsha, 410008 Hunan China

**Publisher Correction to: BMC Biol 19, 200 (2021)**

**https://doi.org/10.1186/s12915-021-01118-w**

Following publication of the original article [1], the authors noted errors in the affiliation list and affiliation assignment due to a typesetting mistake. The correct affiliation list and affiliation assignment is reflected in this Correction article and the original article [1] has been corrected.
