# Supplementary figures and images for: An increase of phosphatidylcholines in follicular fluid implies attenuation of embryo quality on day 3 post-fertilization
Source: BMC Biol. 2021 Sep 9;19:200. doi: 10.1186/s12915-021-01118-w (PMC8428131; doi:10.1186/s12915-021-01118-w)

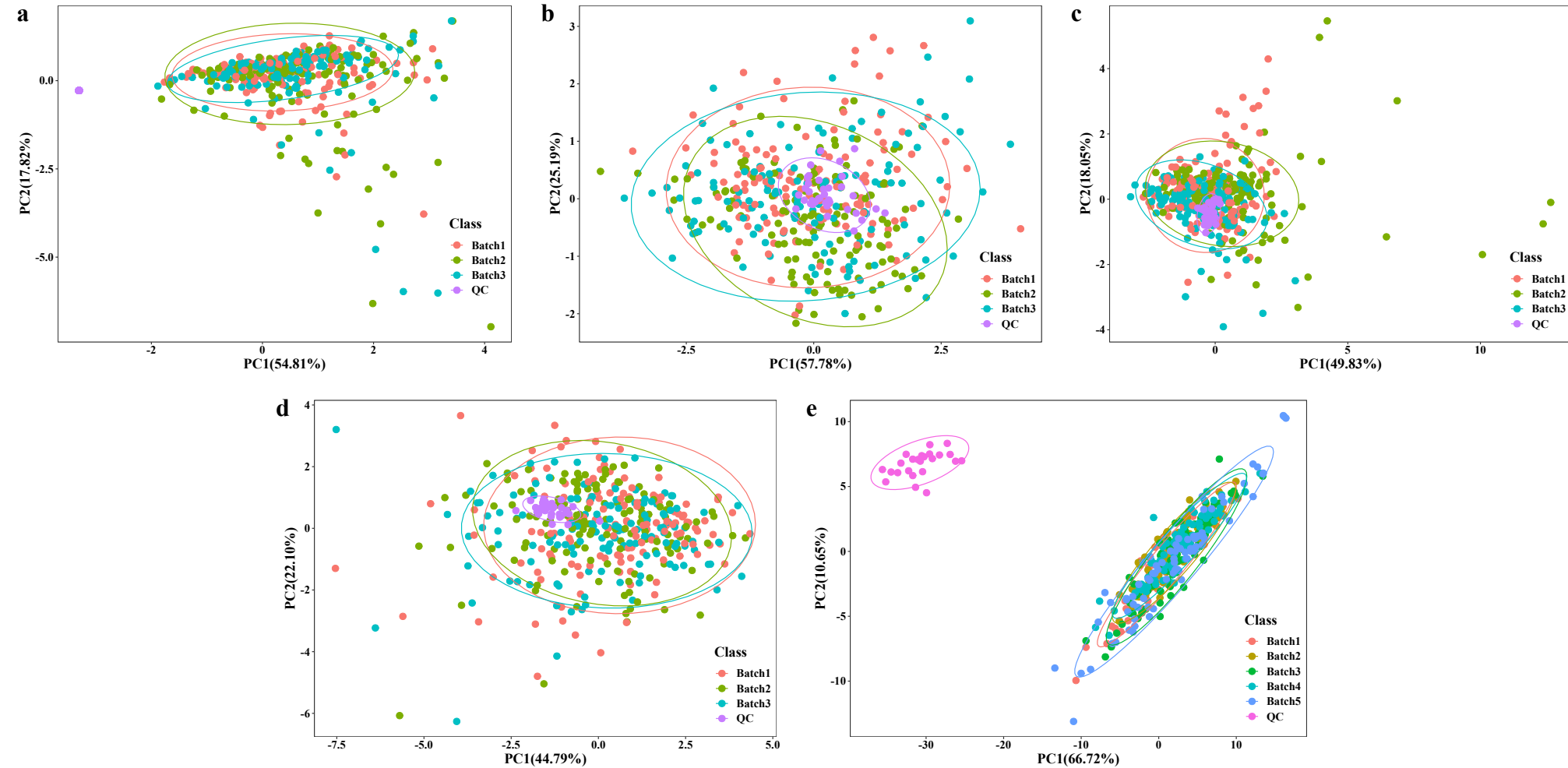

Supplement: Supplementary file 1 — Additional file 1:. Assessment of batch effect with PCA plot in each LC-MS/MS method. The batch effect of amino acid, fat-soluble vitamin, water-soluble vitamin, hormone and P180 method is represented in a-e, respectively. [file 12915_2021_1118_MOESM1_ESM.pdf]

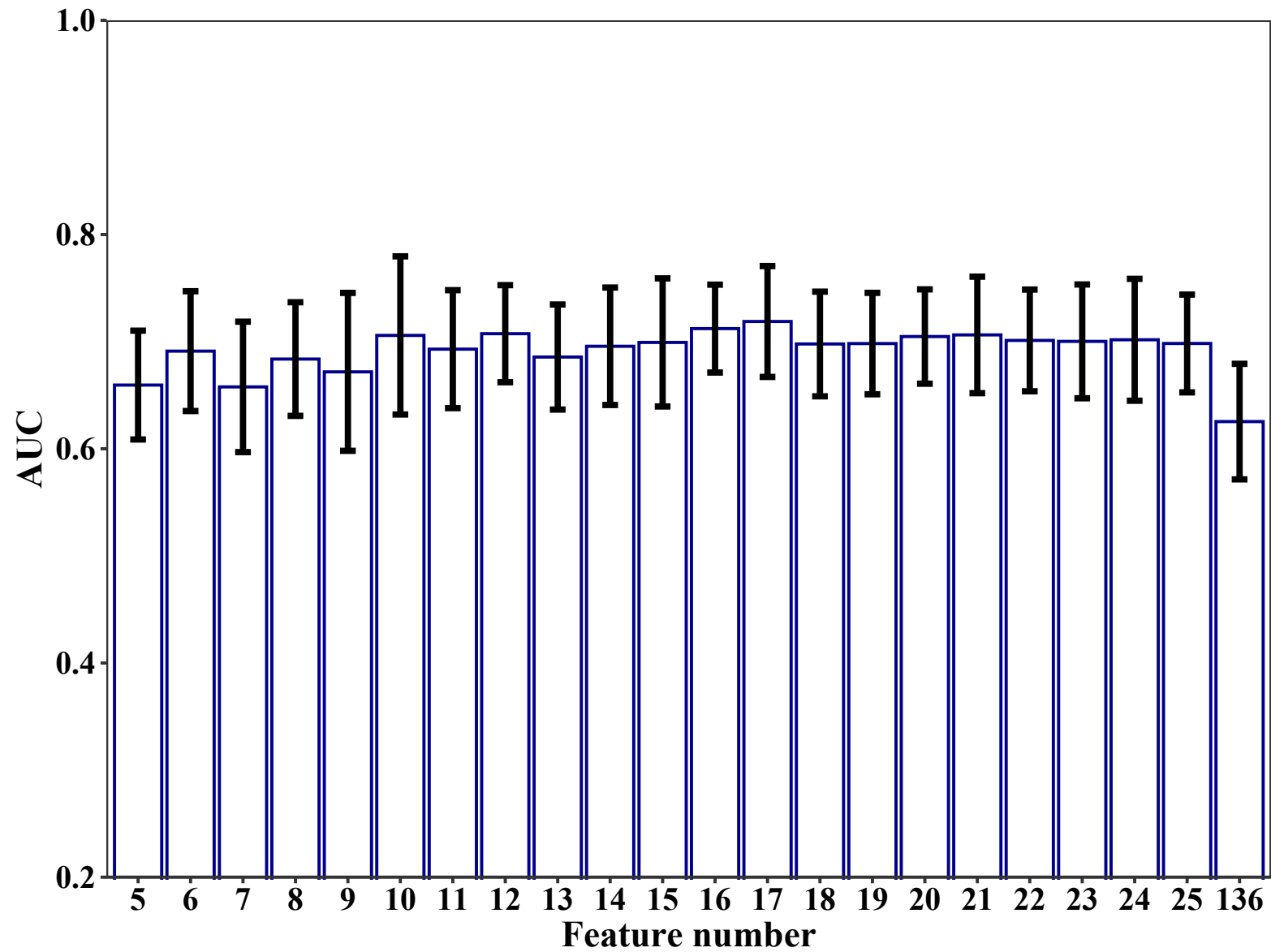

Supplement: Supplementary file 3 — Additional file 3:. Further dimensional reduction of the features consisting of metabolites and clinical parameters rfFuncs. Y-axis represents the average AUC value of ROC in RF models, error bar represents the standard derivation of AUCs, and X-axis denotes the selected features. [file 12915_2021_1118_MOESM3_ESM.pdf]

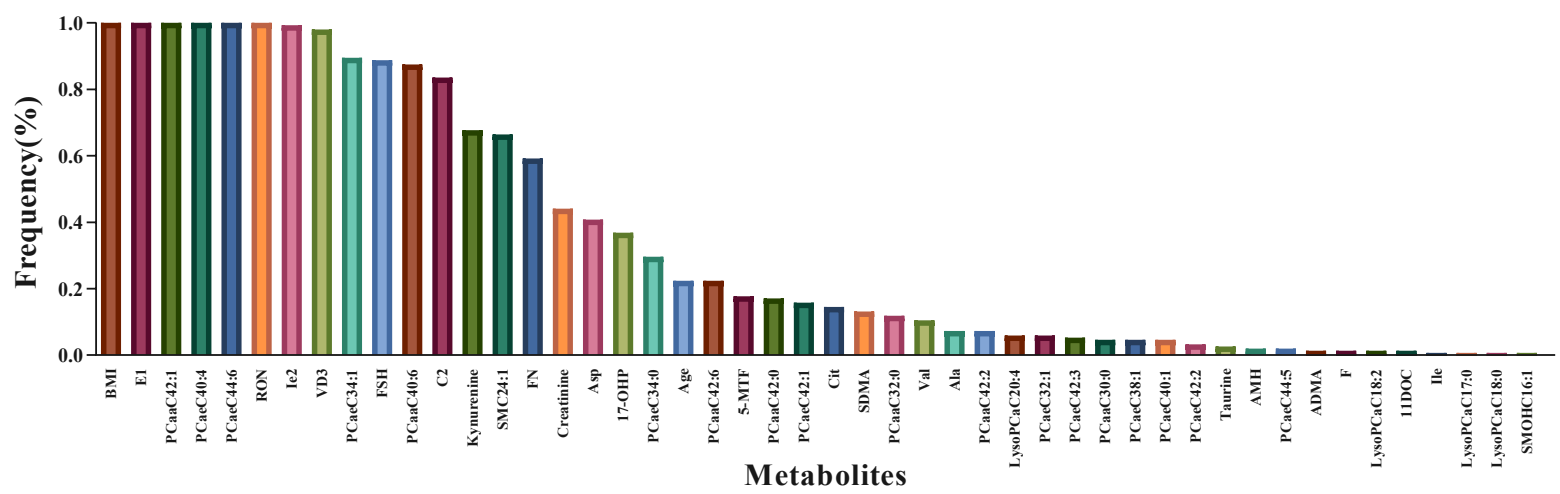

Supplement: Supplementary file 4 — Additional file 4:. The frequency of metabolites selected in 17 features pattern. [file 12915_2021_1118_MOESM4_ESM.pdf]

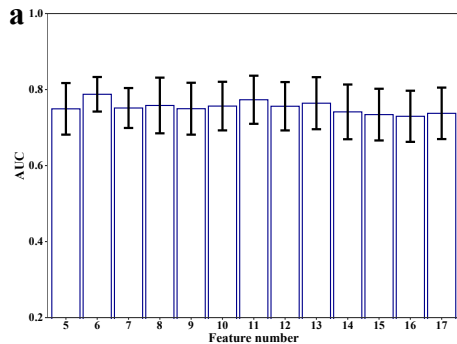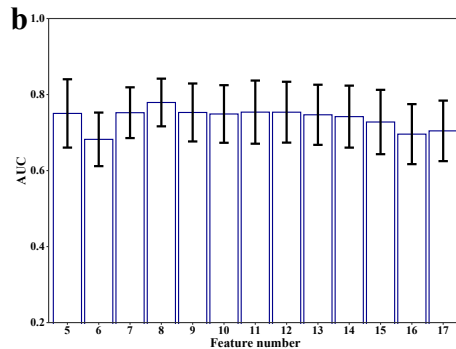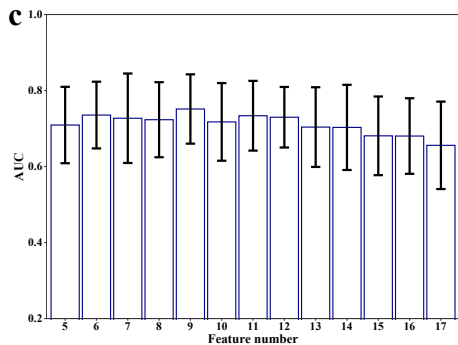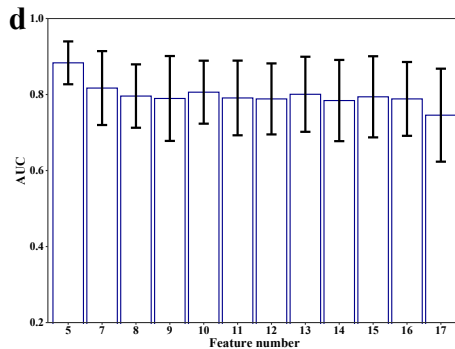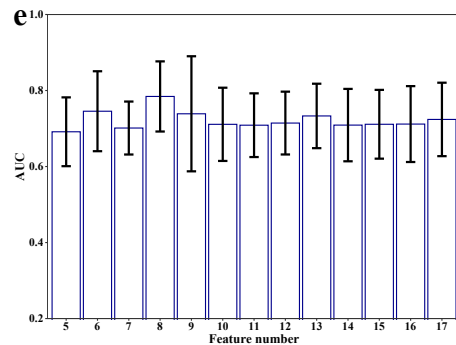

Supplement: Supplementary file 6 — Additional file 6: Further dimensional reduction of the features consisting of metabolites and clinical parameters with rfFuncs in each subgroup. A total of 7 subgroups upon the clinical parameters are selected, (A) the patient ages < 35, (B) the patient ages ≥ 35, (C) the patients received long protocol, (D) the patients received ultralong protocol, (E) the patients received GnRH-ant protocol. The representativeness of X-axis and Y-axis is as same as Figure 3C. [file 12915_2021_1118_MOESM6_ESM.pdf]

**a**

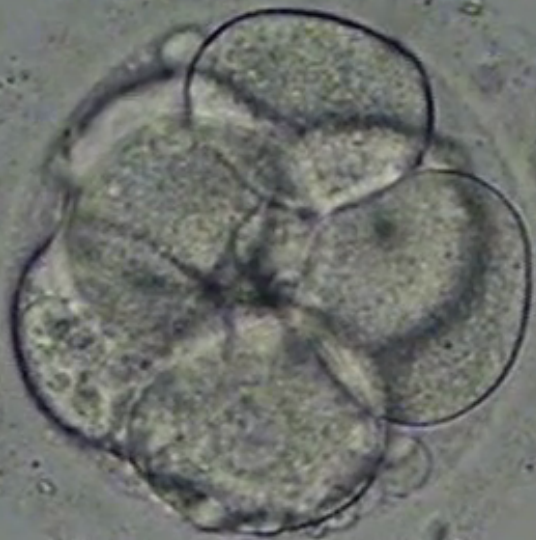

**b**

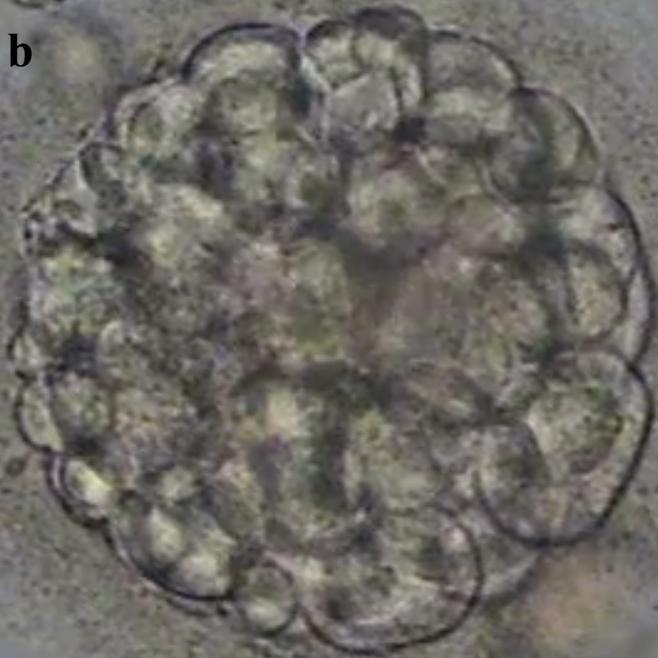

Supplement: Supplementary file 7 — Additional file 7: The morphology of the embryos on day 3 after fertilization. (A) The morphology of the embryo evaluated as G group on day 3 after fertilization (8C-I); (B) The morphology of the embryo evaluated as P group on day 3 after fertilization (IV). [file 12915_2021_1118_MOESM7_ESM.pdf]
