# Supplementary material for: An increase of phosphatidylcholines in follicular fluid implies attenuation of embryo quality on day 3 post-fertilization
Source: BMC Biol. 2021 Sep 9;19:200. doi: 10.1186/s12915-021-01118-w (PMC8428131; doi:10.1186/s12915-021-01118-w)
Supplement: Supplementary file 2 — Additional file 2: Table.S1 Clinical information of the patients. Table.S2 The top 10 AUCs and the corresponding optimal specificities and sensitivities of the metabolites with significant difference between G and P. Table.S3 The sample size in different subgroups. Table.S4 Fold changes of PCs with significant difference between G and P. Table.S5 Elution gradient of all the UPLC methods. [file 12915_2021_1118_MOESM2_ESM.pdf]

Table. S1 Clinical information of the patients

Table. S1(a)

Variate	Overall (mean/SD)	G group (mean/SD)	P group (mean/SD)	P-value (Wilcoxon rank test)	AUC
Age (years)	33.3/5.1	33.5/4.4	33.2/5.7	0.51	0.52
Body mass index (kg/m^2)	22.0/2.5	22.5/2.5	21.6/2.4	0.0010*	0.61
Antral follicle count (n)	15.4/11.7	14.3/9.5	16.3/13.5	0.97	0.50
Retrieved oocytes (n)	6.5/3.2	5.7/2.2	7.2/3.8	0.0011*	0.61
Oocyte recovery (%)	6.5/3.2	85.3/25.3	89.7/30.2	0.33	0.53
AMH (ng/ml)	3.4/3.6	2.99/2.98	3.8/4.0	0.056	0.56
Endometrial thickness（mm）	12.2/2.2	12.3/2.1	12.2/2.3	0.49	0.52
Basic FSH (mIU/ml)	6.2/1.9	6.3/1.6	6.2/2.1	0.15	0.55
Basic LH (mIU/ml)	3.8/2.1	3.7/2.1	3.9/2.2	0.23	0.54
Basic E2 (pg/ml)	48.8/119.1	57.8/171.1	40.6/21.7	0.70	0.51
LH (mIU/ml) on HCG injection day	3.8/2.1	3.7/2.1	2.2/1.9	0.76	0.51
E2 (pg/ml) on the HCG injection day	2176.8/1100.1	1980.1/1039.6	2355.0/1126.1	0.00039*	0.62
Fertilization rate (%)	0.99/0.095	0.98/0.10	0.99/0.087	0.89	0.50
Cleavage rate (%)	0.99/0.051	0.99/0.035	0.98/0.062	0.17	0.52

*Significant at p-value<0.05

Table. S1(b)

Variate	Overall (n)	G group (n)	P group (n)	P-value (Chi-square test)	AUC
Type of infertility				0.25	0.55
Primary infertility	108	44	64		
Secondary Infertility	197	101	96		
Involved with fallopian tube				0.64	0.51
Yes	279	131	148		
No	26	14	12		
Endometriosis				0.64	0.51
Yes	32	17	15		
No	273	128	145		
COH protocol				0.64	
Ultralong	75	33	42		0.52
Long	86	38	48		0.52
GnRH-ant	97	52	45		0.54
Others	47	22	25		0.50
Attempt				0.18	0.56
IVF-ET	161	67	94		
ICSI	144	78	66		

*Significant at p-value<0.05

Table. S2 The top 10 AUCs and the corresponding optimal specificities and sensitivities of the metabolites with significant difference between G and P

Metabolite	AUC	Specificity	Sensitivity
PCaaC42:1	0.638	0.675	0.559
PCaeC44:6	0.636	0.628	0.619
PCaeC40:4	0.625	0.524	0.675
PCaaC40:6	0.617	0.562	0.655
PCaaC42:6	0.615	0.650	0.566
PCaeC34:1	0.615	0.612	0.607
PCaeC42:2	0.613	0.688	0.503
PCaaC38:6	0.612	0.669	0.517
PCaeC40:6	0.612	0.669	0.510
PCaaC42:0	0.611	0.544	0.586

Table.S3 The sample size in different subgroups

Subgroup	Overall (n)	G group (n)	P group (n)	G/P ratio
Subgroup a	181	86	95	0.91
Subgroup b	124	59	65	0.91
Subgroup c	86	38	48	0.79
Subgroup d	75	33	42	0.79
Subgroup e	97	52	45	1.16

Subgroup a-e represent the cohort with age < 35, age ≥ 35, long protocol, ultralong protocol and GnRH-ant protocol, respectively.

Table.S4 Fold changes of PCs with significant difference between G and P

PC	Fold change (P/G)	Adjusted p-value	PC	Fold change (P/G)	Adjusted p-value
PCaaC42:1	1.1551	0.00298	PCaeC34:0	1.103	0.016805
PCaaC40:6	1.1432	0.009666	PCaeC38:0	1.1017	0.029073
PCaeC44:6	1.137	0.00298	PCaaC40:4	1.1015	0.026172
PCaeC32:1	1.1363	0.012265	PCaeC42:2	1.099	0.009666
lysoPCaC20:4	1.1352	0.039805	PCaeC38:6	1.0968	0.03049
PCaaC42:2	1.1279	0.029073	PCaeC38:5	1.0959	0.022419
PCaaC38:6	1.1246	0.009666	PCaeC42:5	1.0907	0.016803
PCaaC36:0	1.1216	0.023064	PCaeC38:4	1.0905	0.025346
PCaaC38:4	1.1214	0.012941	PCaeC34:3	1.0903	0.029073
PCaaC42:6	1.1203	0.009666	PCaeC40:5	1.0898	0.013827
PCaaC42:5	1.1184	0.026172	PCaaC34:1	1.0893	0.029073
PCaeC40:6	1.1139	0.009666	PCaaC32:0	1.0892	0.015402
PCaeC36:5	1.1132	0.02365	PCaeC44:5	1.0874	0.02106
PCaaC38:0	1.112	0.023181	PCaaC30:0	1.085	0.009666
PCaaC36:5	1.1115	0.039805	PCaeC40:2	1.0848	0.026934
PCaaC42:0	1.1111	0.009666	PCaeC36:4	1.084	0.049044
PCaeC42:1	1.1101	0.009666	PCaeC42:3	1.0837	0.012941
PCaeC34:1	1.1092	0.009666	PCaeC32:2	1.0806	0.044026
PCaeC40:1	1.1088	0.018053	PCaeC36:1	1.08	0.018053

PCaaC36:4	1.1071	0.018053	PCaeC36:0	1.0768	0.026934
PCaeC40:4	1.1054	0.006994	PCaeC44:4	1.0759	0.048771
PCaaC38:5	1.1035	0.029073	PCaeC38:2	1.0726	0.036683

Table.S5 Elution gradient of all the UPLC methods

Method	Start time (min)	Flow (ml/min)	Mobile phase A (%)	Mobile phase B (%)
P180 (LC)	0.00-0.72	0.50	100	0
	0.72-5.28	0.50	100	0
	5.28-9.44	0.50	85	15
	9.44-9.68	0.50	30	70
	9.68-9.90	0.50	0	100
	9.90-10.43	0.57	0	100
	10.43-10.72	0.50	0	100
	10.72-12.00	0.50	100	0
P180 (FIA)	0.00-1.60	0.03	0	100
	1.60-2.40	0.03	0	100
	2.40-2.80	0.20	0	100
	2.80-3.00	0.20	0	100
Fat-soluble vitamin	0.00-0.50	0.50	60	40
	0.50-1.50	0.50	20	80
	1.50-2.50	0.50	0	100
	2.50-4.50	0.50	0	100
	4.50-5.50	0.50	60	40
Water-soluble vitamin	0.00-1.00	0.45	99	1
	1.00-1.50	0.45	97	3
	1.50-2.00	0.45	70	30
	2.00-3.50	0.45	30	70
	3.50-4.00	0.45	10	90
	4.00-4.80	0.45	10	90
	4.80-6.00	0.45	99	1
Steroid hormone	0.00-0.90	0.80	75	25
	0.90-1.80	0.80	60	40
	1.80-3.80	0.80	30	70
	3.80-3.90	0.80	5	95
	3.90-5.50	0.80	5	95
	5.50-5.51	0.80	75	25
	5.51-6.50	0.80	75	25
Amino acid	0.00-0.50	0.50	98	2
	0.50-1.50	0.50	90	10
	1.50-3.50	0.50	65	35
	3.50-3.60	0.50	5	95
	3.60-5.00	0.50	5	95
	5.00-6.50	0.50	98	2

P180 (LC): mobile phase A, water (0.2% formic acid), mobile phase B, acetonitrile (0.2% formic acid). P180 (FIA): mobile phase B, methanol (Biocrates solvent I). Fat-soluble vitamin: mobile A, water (0.1% formic acid), mobile phase B, 50% methanol and 50% acetonitrile (0.1% formic acid). Water-soluble vitamin: mobile phase A, water (0.1% formic acid), mobile phase B, methanol (0.1% formic acid). Steroid hormone: mobile phase A, water (1mM ammonium acetate), mobile phase B, methanol (1mM ammonium acetate). Amino acid: mobile phase A, water (1% formic acid and 0.5% heptafluorobutyric acid), mobile phase B, acetonitrile (1% formic acid and 0.5% heptafluorobutyric acid).
