# Supplementary material for: An increase of phosphatidylcholines in follicular fluid implies attenuation of embryo quality on day 3 post-fertilization
Source: BMC Biol. 2021 Sep 9;19:200. doi: 10.1186/s12915-021-01118-w (PMC8428131; doi:10.1186/s12915-021-01118-w)
Supplement: Supplementary file 5 — Additional file 5: A discrimination is relied on 2 statistical evidences, upper panel, ROC analysis, and lower panel, averages of feature importance in a RF model with 17 features. A total of 7 subgroups upon the clinical parameters are selected, (A) the patient ages < 35, (B) the patient ages ≥ 35, (C) the patients received long protocol, (D) the patients received ultralong protocol, (E) the patients received GnRH-ant protocol. The representativeness of X-axis and Y-axis is as same as Figure 3D and E. [file 12915_2021_1118_MOESM5_ESM.pdf]

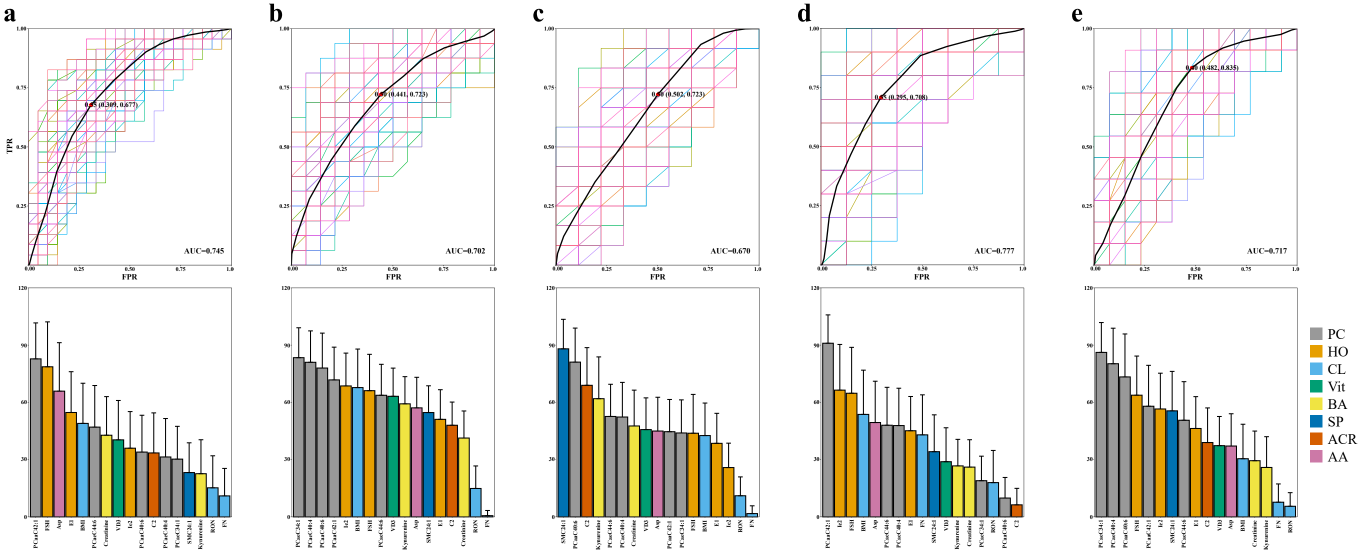

a
b
c
d
e
TPR
FPR
0.55 (0.309, 0.677)
AUC=0.745
0.50 (0.441, 0.723)
AUC=0.702
0.50 (0.502, 0.723)
AUC=0.670
0.55 (0.295, 0.708)
AUC=0.777
0.40 (0.482, 0.835)
AUC=0.717
PCaaC42:1 FSH Asp EI BMI PCaeC44:6 Creatinine VD3 Ie2 PCaeC40:6 C2 PCaeC40:4 PCaeC34:1 SMC24:1 Kynurenine RON FN
PCaeC34:1 PCaeC40:4 PCaeC40:6 PCaeC42:1 Ie2 BMI FSH PCaeC44:6 VD3 Kynurenine Asp SMC24:1 EI C2 Creatinine RON FN
SMC24:1 PCaeC40:6 C2 Kynurenine PCaeC44:6 PCaeC40:4 Creatinine VD3 Asp PCaeC42:1 PCaeC34:1 FSH BMI EI Ie2 RON FN
PCaaC42:1 Ie2 FSH BMI Asp PCaeC44:6 PCaeC40:4 EI FN SMC24:1 VD3 Kynurenine Creatinine PCaeC34:1 RON PCaeC40:6 C2
PCaeC34:1 PCaeC40:4 PCaeC40:6 FSH PCaeC42:1 Ie2 SMC24:1 PCaeC44:6 EI C2 VD3 Asp BMI Creatinine Kynurenine FN RON
PC
HO
CL
Vit
BA
SP
ACR
AA
